# Orthorexia as an Eating Disorder Spectrum—A Review of the Literature

**DOI:** 10.3390/nu16193304

**Published:** 2024-09-29

**Authors:** Izabela Łucka, Artur Mazur, Anna Łucka, Izabela Sarzyńska, Julia Trojniak, Marta Kopańska

**Affiliations:** 1 Department of Developmental Psychiatry, Psychotic Disorders and Old Age Psychiatry, Medical University of Gdansk, 80-282 Gdansk, Poland; izabelalucka@wp.pl; 2 Institute of Medical Science, College of Medical Sciences, University of Rzeszów, ul. Warzywna 1a, 35-215 Rzeszow, Poland; drmazur@poczta.onet.pl; 3 Faculty of Law and Administration, University of Gdansk, 80-309 Gdansk, Poland; lucka.annaa@gmail.com; 4 Student Research Club “Reh-Tech”, Medical College of Rzeszow University, 35-959 Rzeszow, Poland; sarzynskaizabela-2001@wp.pl (I.S.); juliatrojniak0@gmail.com (J.T.); 5 Department of Medical Psychology, Institute of Medical Sciences, Medical College of Rzeszow University, 35-959 Rzeszow, Poland

**Keywords:** orthorexia nervosa, ON, eating disorders, risk factors

## Abstract

Background: The purpose of this study is to compare and analyze research studies focused on orthorexia nervosa (ON) as a spectrum of eating disorders, and to summarize potential risk factors in different age and social groups. ON is characterized by an obsession with healthy eating, which leads to a restrictive diet and health problems. Methods: Due to a lack of comprehensive analyses, this review re-examined studies from 2006 to 2023, initially retrieving 53,134 articles. Upon refining the criteria and risk factors for eating disorders, 34 notable records were identified. These studies employed diagnostic tools such as ORTO and BOT, focusing on risk factors for ON. Results: Results indicate that individuals suffering from eating disorders, losing weight, exercising heavily, developing relationship problems, and suffering from body dysmorphic disorder are at high risk of developing ON. A significant correlation was found between ON, BMI, and gender, but not between ON and OCD. Interestingly, ON symptoms appear to overlap with those of other eating disorders, such as anorexia and bulimia, especially in terms of obsessive control over dieting and fear of gaining weight, indicating a close relationship between the two. Conclusions: Interestingly, orthorexia nervosa may serve as a coping mechanism for anorexia, providing a sense of control. However, further research on its long-term effects is required.

## 1. Introduction

### 1.1. Introduction to Orthorexia Nervosa

In many highly developed countries, a significant increase in harmful eating habits has been observed over the last decade or so. A clear contrast is currently visible in society, as one can meet people affected by malnutrition and obesity. Interestingly, in highly developed countries, “fast food” consumption has increased, but an obsession with healthy eating has also developed. Increasing public awareness of health and sociocultural influences, including social media, have been shown to have a strong impact on eating habits, leading to an increasing incidence of the so-called healthy eating obsession, known as orthorexia nervosa [1].

### 1.2. Historical Context and Definition

The first description of this phenomenon was made by S Bratman in 1997 under the name orthorexia nervosa (ON) [2]. According to Dunn and Bratman, ON is characterized by pathological consumption of healthy food, which in effect leads to nutritional deficiencies and the simultaneous occurrence of tensions in interpersonal relationships [3].

The pursuit of perfect health through diet may result in malnutrition or excessive weight loss. It is common for patients with eating disorders to experience orthorexia nervosa, and research suggests that these symptoms may be present in patients with anorexia nervosa (AN) as well as bulimia nervosa (BN). In contrast to AN and BN, orthorexia nervosa focuses on the quality and purity of food, not its quantity [4]. This concept was coined by Dunn and Bratman and is currently one of the latest formulations that describes ON from a diagnostic perspective. Despite this, ON is still a disease of unknown etiology and epidemiology. Although orthorexia nervosa is increasingly recognized, it has not yet been included in major diagnostic systems such as the *ICD-11* (WHO 2022) and *DSM-5* (American Psychiatric Association 2013).

### 1.3. Prevalence and Risk Factors

As with other eating disorders, ON occurs frequently. A large meta-analysis comprising 30,476 attendees from 18 countries determined the global prevalence of orthorexia nervosa symptoms at about 30% and affects both men and women equally [5]. Research shows that adolescents and young adults are particularly susceptible to orthorexic tendencies due to the influence of social media and peer pressure. Nearly 49% of young adults exhibit symptoms of orthorexia nervosa, driven by idealized images of healthy eating promoted online [6]. Furthermore, studies indicate that the prevalence of orthorexia nervosa among medical students can reach up to 76.2%, as they tend to focus more on health and body image [7]. Adolescents aged 15–21 also display significant orthorexic behaviors, with average ORTO-15 scores around 39.2, highlighting the impact of social and academic pressures [8]. These figures demonstrate that orthorexia nervosa is not evenly distributed across all age groups but is notably higher among younger populations, especially those engaged in health-related fields or exposed to media promoting dietary ideals.

The overall percentage was higher in groups of athletes or fitness enthusiasts. Also intriguing is that orthorexia nervosa symptoms seem to be increasing over time [5].

### 1.4. Challenges in Classification and Diagnosis

Recently, research on orthorexia nervosa (ON) has grown in popularity because of the increasing number of reported cases [9]. Scientific literature lacking a standardized definition of orthorexia nervosa (ON) makes it hard to compare studies and identify risk factors, pathophysiology, and effective treatments [10]. Research is ongoing on this relatively new manifestation of eating disorders, with some researchers considering it an eating disorder, others postulating that it should be classified as obsessive–compulsive disorder, and there are also those who wonder whether ON belongs to behavioral addictions. However, there is still insufficient empirical evidence to support the recognition of ON as a distinct disorder, which leaves it in limbo. This is influenced by the lack of consensus among researchers regarding the definition and classification of ON. Because orthorexia nervosa has symptoms that overlap with anorexia nervosa (AN), bulimia nervosa (BN), and obsessive–compulsive disorder (OCD), it is difficult to classify. The absence of a clear definition makes it difficult to determine whether ON is a distinct condition or a subset of other disorders such as anorexia nervosa (AN), avoidant-restrictive food intake disorder (ARFID) or obsessive–compulsive disorder (OCD) [10].

The orthorexia nervosa (ON) is a growing eating disorder characterized by an unhealthy obsession with eating only clean, healthy foods, which can lead to malnutrition and social problems. Research and diagnosis are challenging due to the lack of a standardized definition and ongoing debate regarding whether ON is a separate disorder or part of other conditions such as anorexia or obsessive–compulsive disorder. The state of affairs suggests that previous findings and research results should be systematized for the purpose of synthesizing the findings. Consequently, in our review, we reanalyzed and reviewed published research.

With a focus on risk factors and questionnaire tools for ON identification, we selected 34 articles by reviewing the MEDLINE/PubMed, Wiley Online Library, SpringerLink, and Scopus databases. The search criteria were narrowed by eliminating articles that did not include current diagnostic tools; studies with flawed methodology, e.g., studies with healthy participants only, too small study groups, or biased selection of participants; as well as studies that used a healthy diet as a condition for treating disease.

## 2. Methodology and Selection Criteria

Our article reviews the literature of publications published between 2006 and 2023. The analysis was performed using the following search engines: MEDLINE/PubMed, Wiley Online Library, SpringerLink, and Scopus. Our review focused on clinical studies that examined risk factors for orthorexia nervosa and its potential classification as an eating disorder. By searching PubMed using the keywords “eating disorders”, we were able to identify 52,781 articles, which were further refined by including additional keywords such as “orthorexia nervosa”, “diagnostic scales”, and “risk factors”. Additionally, filters were utilized in order to limit the results to empirical studies, leading to the selection of 34 articles that were relevant to the study. Then, focusing exclusively on our topic—orthorexia nervosa—38 scientific texts were extracted. One search engine, the Wiley Online Library, found 97 publications, 6 of which were about ON and the prevalence of risk factors. A total of 256 articles were retrieved from SpringerLink’s resources, of which 21 met our requirements. When searching for relevant publications, we did not take into account collective studies.

Several factors may be contributing to the reduction in articles available in some databases, including changes in policy, changes in publication focus, or limitations in the inclusion criteria. It is possible that these limitations impacted the comprehensiveness of the literature review, potentially limiting its scope. However, we ensured that the available resources were exhaustively analyzed to minimize any impact on the review’s robustness.

We initially selected 65 articles published between 2006 and 2023 that examined prevalence and risk factors for orthorexia and other eating disorders, based on titles and abstracts. A further analysis in terms of methodology and results was performed by reading the complete texts of 56 of the originally selected articles, which were then entered into the model. As exclusion criteria, after careful analysis, we used studies with methodological flaws—e.g., studies with healthy participants only; too small study groups, which we considered fewer than 30 study participants; or biased selection of participants—and publications that did not include the questionnaire tools currently used to identify ON. Studies that included only healthy participants and those focusing on mindfulness practices were excluded in accordance with the scope of the review. Although orthorexia nervosa (ON) is associated with mental health and emotional regulation, this study aimed to examine pathological behaviors linked to obsessive healthy eating. Although mindfulness practices are beneficial in many mental health contexts, they are incompatible with the compulsive nature of ON, and therefore were excluded in order to maintain clarity in the distinction between therapeutic practices and harmful behaviors.

We noted that the most popular questionnaires were the ORTO, the BOT (Bratman Test for Orthorexia), the Treuel Orthorexia Scale, and the Dusseldorf Orthorexia Scale. These orthorexia nervosa tests were developed for the purpose of measuring tendency and symptoms related to the obsession with healthy eating. While all of these scales are utilized to identify orthorexia nervosa, their precision, popularity, and research scope differ. There are two main limitations to these tools: a lack of clear diagnostic criteria and difficulty in distinguishing orthorexia nervosa from healthy eating habits.

We also did not take into account articles in which the study group consisted of people for whom healthy nutrition was one of the conditions for effective treatment of various diseases, e.g., postpartum mothers and persons with diseases related to the nervous system such as diseases of the intestinal tract. We excluded groups such as postpartum women and patients with neurological conditions due to the possibility that their health status could cause confounding effects on their dietary behaviors. In many cases, these groups require specialized nutritional plans in order to recover or treat their conditions, which may not be reflected in their orthorexia tendencies. It is possible that their inclusion could distort our understanding of ON since it specifically addresses pathological eating behaviors in otherwise healthy individuals.

From the remaining works, we excluded studies that described the positive impact of mindfulness practices on health. In our review, we analyzed 34 remaining works.

**Figure 1:**
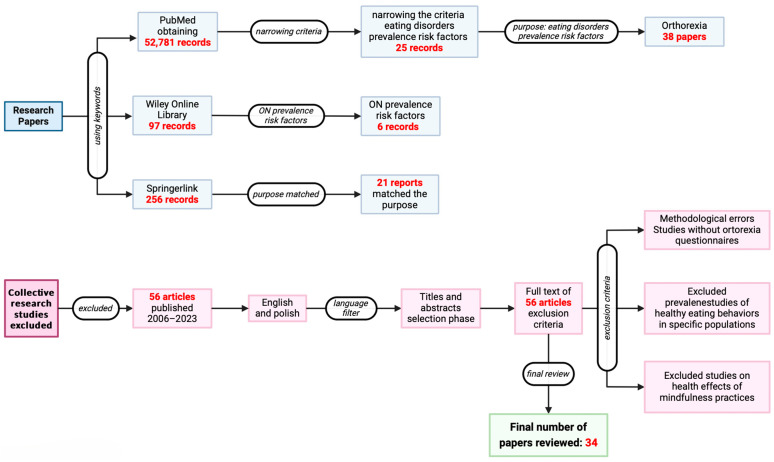
A diagram of the choice-making process.

The set-up process is described in the schematic below—Figure 1.

As a point of clarification, it is important to note that diagnostic tools used to assess orthorexia nervosa, such as the ORTO-15, have known limitations, especially in the area of distinguishing ON from healthy eating habits. Furthermore, this review had some limitations, including difficulty finding current studies and comparing results across studies that used different orthorexia diagnostic scales.

## 3. Overview of the Research Review

### 3.1. Prevalence Studies

In our review, we finally analyzed 21,341 male and female subjects. After analyzing the selected publications, we wanted to estimate the prevalence of orthorexia nervosa and identify possible causes of its occurrence. The limited number of publications meeting the required criteria makes it difficult to achieve our goal. The reason is that the selected articles analyze different groups of patients; some of the works concern population studies, while others precisely specific groups, e.g., people with self-identified eating problems, athletes, creatives, or people addicted to social media. Interestingly, the authors emphasize the exceptionally high frequency of orthorexia among social media users (90.6%) because these results may distort the overall picture of ON by overrepresenting this subgroup compared to the general population. The high values obtained in this group may result from the fact that the analyzed works used a variety of survey forms, which results from the fact that the diagnostic process of ON has not yet been precisely standardized. This diversity is an obstacle to obtaining potentially objective results. In some studies, using the ORTO-15 questionnaire, the threshold is 40. As a result, other researchers using this threshold obtained significantly overestimated results suggesting the estimated prevalence of orthorexia nervosa. Using this cut-off point, the prevalence rate even reached 86%, with a range from 56.4% to 90.6%. Therefore, it can be seen that when using the ORTO-15 questionnaire, it is considered correct to use 35 as a cut-off point [11,12,13,14]. After discarding excessively high scores and considering the use of other ORTO/BOT (Bratman Test for Orthorexia) tests, it can be concluded that the prevalence rate of orthorexia nervosa oscillates between 6.5% and 41.7%, according to various studies. Thus, it can be concluded that an average of 24% of patients suffer from orthorexia nervosa. Interestingly, the highest likelihood of ON is found in the population of people with disorders related to poor eating, such as anorexia and bulimia [13,15,16,17,18,19,20,21,22,23,24]—Table 1.

### 3.2. Personality Traits and Orthorexia

Among the analyzed studies, the most interesting are the works in which the main topic was the study of the relationship between personality traits and the development of orthorexia nervosa. The greatest influences on morbidity were obsession with body image, dieting, obsessive focus on concerns about looks and weight, combined with difficulties in the process of fitting into the changing environments, implementation of yet unseen defense mechanisms, and a low rate of self-acceptance [20,21,23,25,26,27,28]. The aforementioned features are also found in people suffering from other eating disorders. They appear to be regularly observed in all people affected by eating disorders. Interestingly, other researchers have shown that people suffering from OD and ED are characterized by problems with emotion regulation and identification. The authors also observed that people with ON correctly characterize the emotions they experience [20].

### 3.3. Orthorexia and Related Eating Disorders

Other studies show a correlation concerning ON and anorexia nervosa (AN). It can be stated that the manifestations of orthorexia nervosa are similar to those observed in anorexia nervosa; these are perfectionism, excessive physical exertion, problems in relationships, and an attitude towards nutrition that is perceived by patients as a way to feel dominance over themselves and their lives [12,18,20,24,29,30,31,32,33,34,35,36,37,38]. It is very interesting that ON patients have abnormal attachment styles. For this reason, ON should be classified as an eating disorder in the future. In our work, we want to propose an interpretation of ON as a specific type of ED. Many investigators note a lot of correlations between the occurrence of ON and dysfunctions regarding personality, various symptomatic features, and the everyday capability of patients. Subsequent publications show the relationship occurring the interplay between ON and BMI. The analyzed studies included a group of 5048 people. In 1312, a statistically significant relationship between high BMI and orthorexia nervosa was observed. The publication, in which the research group consisted of 1120 people, did not show such correlations [14,39,40]—Table 2. On the other hand, three studies, in which a total of 2616 people participated, showed a quantitatively significant association between low BMI and orthorexia nervosa [41,42,43]. The obtained results are very interesting, but they require in-depth research and more detailed analysis. Such studies can be enriched with imaging diagnostics and more precise screening tests, which will reveal people affected by disorders and people at risk for this type of condition.

In the studies analyzed, it can be noted that ON patients are characterized by perfectionism, appearance concerns and interpersonal difficulties. The authors of the analyzed articles did not focus on a detailed analysis of the correlations between these factors. Further research in this area is needed to get a more accurate picture of these correlations and their impact on the incidence of ON.

However, interestingly, similar correlations are observed in people suffering from anorexia. Researchers show that a hallmark of anorexia patients is the presence of high levels of perfectionism, which in effect influences critical and harsh self-assessment [44]. In addition, people with anorexia often face problems in interpersonal relationships, which may be a result of their high expectations of themselves [45]. This comparison may provide valuable insights into the complex interactions between risk factors that are equally common in ON patients. In the case of the frequency of orthorexia nervosa by gender, the authors of the study observed a correlation between the female gender and the occurrence of ON [40]. It is also noteworthy that in three studies, in which 1254 people participated, the authors, focusing on the diagnostic classification of the orthorexia nervosa phenomenon, did not observe a relationship between ON and obsessive–compulsive disorder [13,24,41].

**Table 2:** Other risk contributors (personality traits, body image, excessive exercising, eating habits, BMI, gender).

No.	Researcher	Purpose of the Survey	Research Methods	Study Group		Frequency	Results and Comments
				Number of Participants	Group Characteristics		
1	[26]	Assessing the prevalence of orthorexia nervosa and the different causes of orthorexia nervosa in Austrian female nutritionists.	FEV ^1^ assessment, BOT ^2^ evaluation	283	The group consisted of Austrian women aged 22–66 years who were dietitians	12.8% of female participants showed symptoms of orthorexia nervosa	Stress, emotional crisis, and serious emotional and physical distress. These results suggest that orthorexia nervosa is a relatively common eating disorder among dieters.
2	[27]	The study shows the importance of understanding the effects of previous family experiences with ED ^3^	Tugrul scale, EAT-26 ^4^, Teruel Orthorexia Nervosa Scale (TOS) ^5^	225	The study group consisted of adults aged 18–24 years old	“Healthy orthorexia nervosa” (HO) ^6^ and “diet-related disorders” (HEF) ^7^ had a significant impact on participants’ eating attitudes. The association between eating attitudes and HEF ^7^ was r = 0.57	Orthorexia nervosa in healthy individuals may result from limited social activities and family problems.
3	[23]	Investigating whether mental anorexia correlates with ON ^8^	ONI ^9^, BRIEF-A ^10^	405	Among participants, 80% were female. Half Caucasian. Average age = 24 years, average BMI = 25	The study found that orthorexia nervosa was weakly to moderately correlated with most BRIEF-A scales, with the most correlated with behavioral regulation, such as emotional control (r = 0.34) and inhibition (r = 0.30)	Anorexia and orthorexia nervosa have similar symptoms and share the same neuropsychological nature.
4	[25]	Investigate the associations between ON ^8^ and EDs ^3^, psychopathological symptoms, and defense mechanisms	ON (EHQ-21) ^11^, eating psychopathology (EDI-3) ^12^, psychopathological symptoms (BSI) ^13^, defense mechanisms (DSQ-40) ^14^	270	Students from the University of Palermo in southern Italy and were assigned to three groups: 52 people with ON ^8^ symptoms, 157 people constituted a healthy eating control group, and 61 people following a normal diet	Not specified	ON ^8^ associated with more frequent psychopathological episodes and the use of various pathological defense strategies.
5	[28]	The aim was to find out examination of the risks of developing ON ^8^ with emphasis on individual factors	Answered self-administered questionnaires assessing ON ^8^	3235	The study group consisted of 10.32% men and 89.67% women, with a mean age of 21.13 years. Participants self-completed questionnaires assessing orthorexia nervosa (ON) ^8^ and other	In a study conducted on the same random population, it was discovered that orthorexia nervosa (ON) ^8^ was exhibited by 3.28% of the participants. The mean F-DOS ^15^ scale score for the whole group was 19.2 (SD = 4.95), while in the orthorexic group the mean score was 32.62 (SD = 2.27)	ON ^8^ can collude with different character models, and some of them are characterized by a significant level of psychopathology.
6	[21]	The aim was to measure orthorexia using an assessment method	Temperament Character Inventory-56 (TCI-56) ^16^, Ortho-11-Hu ^17^	739	Participants’ ages ranged from 18 to 72 years. The research group consisted of doctors, athletes, nutritionists, performance artists, and Ashtanga yoga practitioners	The mean ORTO-11-Hu ^17^ score was 22.71 (SD = 4.55). The high ON tendency group (score 14–29) comprised 234 subjects and the low ON ^8^ tendency group (score 35–43) comprised 245 subjects. Women were more likely than men to show high ON ^8^ tendencies	Psychological indicators of AN ^18^ and BN ^19^ indicate the following important parameters of orthorexia nervosa.
7	[20]	Investigate whether ON ^8^ correlates with anorexia and bulimia in relation to perfectionism, attitudes towards body image and attachment style	ORTO-15 ^20^, MPS ^21^, MBSRQ-AS ^22^, RSQ ^23^, RSES ^24^	220	The survey group was 46 men and 174 women; 180 of those were first- and second-year psychology students at James Cook University	The mean score for ORTO-15 (based on nine items) was 22.71 (SD = 4.55). There was no significant difference in ORTO-15 ^20^ scores between men and women	Stronger orthorexic tendencies were significantly correlated with incidences of perfectionism.
8	[18]	Analysis of possible links the relationship of ON and psychological behaviors and functions that are widespread to EDs ^3^	ORTO-11-ES ^26^, EDI-2 ^27^	492	The research group consisted of students from the University of Castilla-La Mancha in Spain (280 young women and 212 men)	Confirmatory factor analysis (CFA) ^25^ identified 11 items and 3 attributes as the superior matching model; CFI ^28^ = 0.94, TLI ^29^ = 0.91, RMSEA ^30^ = 0.058	Many mental and organizational behavioral issues of the erectile impairment are shared by those at increased risk of ON ^8^.
9	[46]	The aim was to explore the relationship between inappropriate spatial pattern of social behavior and coping strategies	ON (ORTO-15 ^20^—reduced to ORTO-7CS), EAT-26 ^4^, alexithymia (TAS-20) ^31^, emotion dysregulation (DERS-16) ^32^	196	The study involved167 women, 29 men. The age range was 18–66 years	Not specified	Problems with recognition and control of experienced emotions are associated with higher rates of ON ^8^.
10	[41]	Assessment of the occurrence of ON ^8^ in people with disordered eating behavior and body image in the study group	Eating Attitudes Test-26, the Body Shape Questionnaire-34, ORTO-15 ^20^	92	Participants are adult Australians recruited at the university	The prevalence rate of orthorexia nervosa was 21% using an ORTO-15 ^20^ cut-off value of <35	Significant risk factors for ON ^8^ include being overweight, being unhappy with body image, and challenges in terms of verbal skills.
11	[29]	Investigating the relationship between ORTO-15 ^20^ score and test for obsessive–compulsive symptoms, eating disorders, and body-related discomfort.	ORTO-15 ^20^ test, the Maudsley Obsessive–Compulsive Questionnaire, the Eating Attitudes Test-26, the Body Uneasiness Test	120	Students currently signed up for the first year of their medical studies and master’s degrees in literature and philosophy at the University of Pavia, of which there were 83 women and 37 men	In female students, a statistically relevant, though weak, correlation was observed between ORTO-15 ^20^ and body image discomfort (r = 0.39)	Lower the ORTO-15 ^20^ scores the less pathological body image discomfort and obsessive–compulsive symptoms.
12	[30]	Study aimed to identify the relationships between orthorexia nervosa and social appearance anxiety	Orthorexia Nervosa Scale	430	215 nursing students and 215 physical education students	Among students in the Faculty of Sport Sciences (FSS), orthorexia nervosa was present in 28.8% of cases, while in the Faculty of Nursing (ND) it was 16.3%	There was a negative relationship between scale SAAS ^33^ and RSES ^24^, which are considered determinants of ON ^8^.
13	[31]	Study examined the prospective associations between five components bodily perceptions and manifestations of ON ^8^ in adult women in a social context	Other	558	Women from the worldwide population	Not specified	Negative body image might be implicated in the onset or maintenance of ON ^8^ symptoms.
14	[18]	The prevalence of ON ^8^ and to analyze potential links between ON ^8^ and psychological traits frequently observed in eating disorders (EDs) ^3^	The ORTO-11-ES ^26^ questionnaire, the Eating Disorder Inventory (EDI-2) ^27^	454	The research group consisted of students from the University of Castilla-La Mancha in Spain (280 young women and 212 men)	Confirmatory factor analysis (CFA) identified 11 items and 3 attributes as the superior matching model; CF ^28^ I = 0.94, TLI ^29^ = 0.91, RMSEA ^30^ = 0.058	The results of the EDI-2 ^28^ study in the ON ^8^ exposure group seemed to suggest that the specific characteristics of individuals were associated with a higher risk of ON ^8^.
15	[32]	Evaluation of the relationship between religiosity and orthorexia nervosa	Teruel Orthorexia Nervosa	428	Participants were individuals over the age of 18 with Lebanese citizenship	Not specified	The level of self-esteem was associated with low levels of mental orthorexia nervosa. Higher religiosity was shown to be associated with higher self-esteem.
16	[24]	This study examined how used test scores correlate with BOT ^2^ scores in relation to age, gender, and self-reported exercise duration.	Sociocultural Attitudes Towards Appearance Questionnaire (SATAQ) relate to Bratman’s orthorexia nervosa test (BOT)	251	The research group consists of people attending fitness classes in five fitness centers in a city in southwest Sweden	The prevalence of orthorexia nervosa (ON) ^8^ in the men’s and women’s study was determined by different analytical models	Women’s gym participants who exercised more frequently had high BOT scores. This validates the link between increased exercise and problematic weight management behaviors, as well as ED ^3^.
17	[33]	The goal was to scrutinize the connection between exercise addiction EA and ON ^8^	Compulsive Exercise Disorder Inventory (EAI) ^34^, Düsseldorfer Orthorexie Skala (DOS)	1008	The group consisted of 559 male and 449 female active members of three fitness studios	In the study group, 10.2% have symptoms of anorexia and 3.4% suffer from orthorexia nervosa. In addition, 2.3% of participants struggle with both disorders simultaneously	Exercise addiction and excessive exercising connected with more pronounced ON ^8^.
18	[12]	The focus was on investigating the frequency of ON ^8^ in university students who take part in various competitions	ORTO-15 ^20^	215	The study group was aged between 20 and 22 from universities in North East England who had completed the ORTO-15 test (≤40)	ON ^8^ symptoms were high in all students (76%)	Students and especially those who take part in university competitions can get sick from ON ^8^.
19	[34]	Ashtanga yoga is a predisposition to ON ^8^	ORTO-15 ^20^	136	The research thicket was the local Ashtanga yoga community	In a study of 136 people, the mean score on the ORTO-15 ^20^ test was 35.27 ± 3.69. As many as 86% of participants scored below 40	Ashtanga yoga predisposes ON ^8^. The results of the study suggest that an intense focus on a healthy diet, which is often promoted in the context of Ashtanga yoga practice, may lead to the development of orthorexia nervosa (ON) ^8^.
20	[35]	The aim of the study was to assess the prevalence and some psychological and other correlates of ON ^8^ tendencies among gym-goers	(Orto-11-Hu) ^17^ and independent variables (Eating Disorder Inventory, Maudsley Obsessional–Compulsive Inventory, health and exercise habits, and demographics)	207	The participants were gym-goers. The average age was about 32 years. Most were women from higher level of education	The mean score on the ORTO-11-Hu test was 27.7. The propensity to ON ^8^ was more prevalent in those who strived for an ideal body shape	Results suggest that ON ^8^ is associated with frequent exercise and younger age.
21	[36]	The aim was to study the relationship between vegetarianism and orthorexia nervosa (ON) ^8^	General characteristics, anthropometric data, the Bratman Test for Orthorexia nervosa (BOT) ^2^, and questions assessing attitudes toward food and nutrition	2611	1346 vegetarians and 1265 non-vegetarians	The incidence of mania for healthy food is more common among vegetarians than non-vegetarians	Intense attachment to healthy food is higher in vegetarians, especially lactovegetarians, and ON ^8^ prevalence decreases with age.
22	[37]	Analysis to determine if ON ^8^ risk factors are related to lifestyle	ORTO-15 ^20^	671	Participants in the study were students at the University of Pavia. Participants were 654% female, 46% male	The results of the ORTO-15 ^20^ test showed that 31.2% of participants scored below 35, indicating a risk of orthorexia nervosa	Diet is a major determinant of ON ^8^.
23	[38]	The aim of the study was to assess the prevalence of ON ^8^ symptoms in organic shop customers and compare the results those of with patients without OSC ^35^	ORTO-15 ^20^ Eating Habits Questionnaire (EHQ)	121	The research group was customers of organic shops	Prevalence among OSC was 69.4% and 23.1% using ORTO-15 ^20^ with 40 and 35 cut-off points	Organic store customers (OSCs) ^35^ may be a population at risk for ON ^8^.
24	[47]	The aim of the study was assessment of the frequency of ON ^8^ and association with the sex and dietary habits in relation to early adulthood	ORTO-15 ^20^	2130	Participants were University of Pisa students who anonymously completed the ORTO-15 ^20^ questionnaire and a form on sociodemographic characteristics and eating habits.	The mean ORTO-15 ^20^ score was 36.93 ± 4.22, with 34.9% of participants scoring below 35, indicating a risk of orthorexia nervosa	Higher ON ^8^ rates in women (37.8% vs. 30.7%), vegans/vegetarians (56.3% vs. 32.2%), and those with a low BMI (42.8% vs. 34.2%).
25	[48]	The aim was to explore problematic eating behaviors in a vegan population	Scales in Orthorexia nervosa, Self-Compassion, Mindful, Emotional, External, and Restraint Eating	315	The group consisted of 287 women and 28 men who followed a vegan diet and completed orthorexia nervosa tests	The prevalence of orthorexia nervosa in the study group was determined by correlation analysis	People with high levels of ON ^8^ show low levels of self-compassion and high levels of restrained eating.
26	[49]	The investigation focused on the relationship between ME and perfectionism in the etiology	Düsseldorf Orthorexia nervosa scale, the Mindful Eating Behavior scale, the Big-Three Perfectionism scale Short-form	670	The study group consisted of 588 women and 78 men (*n* = 670), and the age of the participants ranged from 18 to 74 years.	Not specified	A significant relationship was found between the “aware” aspect of ON ^8^. People suffering from perfectionism are in the ON risk group.
27	[14]	Prevalence and risk factors of ON	ORTO-15 ^20^, EAT-26 ^4^, MOCI ^36^, BDI-II ^37^, individual questionnaire	864	599 women aged 17 to 23 and 265 men aged 15 to 21	27%	Overweight 13–16-year-olds are more likely to suffer from ON ^8^.
28	[39]	Test BOT ^2^ for ON ^8^ diagnosis, and its relationship to validated tools for assessing disordered eating	BOT ^2^, EAT-26 ^4^, BDDQ ^38^, OCI-R ^39^	448	The group was made up of college students with an average age of 22 years old	Significant positive correlations were observed between ON scores and total BOT scores (r = 0.47, *p* < 0.01), EAT-26 (r = 0.25, *p* < 0.01) and BDDQ (r = 0.19, *p* < 0.01)	Hispanic/Latino and overweight/obese as a predisposition for ON ^8^.
29	[47]	The “awareness” facet demonstrated a significant link with ON ^8^, and individuals with perfectionistic traits are more likely to be at risk for ON	ORTO-15 ^20^	2130	Participants were University of Pisa w students who anonymously completed the ORTO-15 ^20^ questionnaire and a form on sociodemographic characteristics and eating habits.	34.9%	ON rates were considerably higher among those with younger BMI values.
30	[41]	The aim is to estimate the prevalence of ON ^8^ and identify risk factors for orthorexia nervosa	Eating Attitudes Test-26, the Body Shape Questionnaire-34, ORTO-15 ^20^	92	Participants are adult Australians recruited at the university	The prevalence rate of orthorexia nervosa was 21% using an ORTO-15 ^20^ cut-off value of < 35	Key contributors to ON risk are being underweight, having issues with body appearance, and experiencing poor social functioning.
31	[40]	To investigate the frequency of ON in relation to dietary behavior and body satisfaction among college undergraduates.	ORTO-15, BPPPS ^40^, FFQ-6 ^41^	1120	Health and non-health students from seven universities in Poland	When the cut-off point was 40, a tendency towards orthorexia nervosa (ON) was shown by 75.0% of participants. When the cut-off point was 35, the prevalence of ON was equal to (28.3%)	Health students at increased exposure to high risk of ON ^8^.
32	[50]	Study to identify demographic factors and predictors of ON, including PrEP use, online portals, and Grindr ^®^	ORTO-15 ^20^, EAT-26 ^4^	394	Participants were gay men aged 18 and over who identified themselves as male. There were 188 participants from Poland and 206 from Spain	Orthorexia nervosa was found in 40% of Polish participants, compared to 28% of Spanish participants	The most important predictors of mental anorexia in homosexual men are low BMI and use of Grindr.
33	[47]	The analysis was to demonstrate the frequency of ON and its relationship with gender and dietary style among participants	ORTO-15 ^20^	2130	Participants were University of Pisa w students who anonymously completed the ORTO-15 ^20^ questionnaire and a form on sociodemographic characteristics and eating habits.	The mean ORTO-15 ^20^ score was 36.93 ± 4.22, with 34.9% of participants scoring below 35, indicating a risk of orthorexia nervosa	Significantly higher rate of ON ^8^ in women than in men (37.8 vs. 30.7%).
34	[51]	The study aimed to establish the relationship between eating problems, mental anorexia, gender and BMI, and field of study among Turkish female students	EAT-40 ^42^, ORTO-15 ^20^	900	The research group was students aged 17–23	The proportion of orthorexia nervosa female subjects is higher than the number of males	Women are more likely to have ON ^8^.

^1^ FEV Assessment—Food-Related Experience and Behaviors Assessment; ^2^ BOT—Bratman Orthorexia Test; ^3^ ED—eating disorder; ^4^ EAT-26—Eating Attitudes Test-26; ^5^ TOS—Teruel Orthorexia Nervosa Scale; ^6^ HO—healthy orthorexia; ^7^ HEF—diet-related disorder (health eating fixation); ^8^ ON—orthorexia nervosa; ^9^ ONI—Orthorexia Nervosa Inventory; ^10^ BRIEF-A—Behavior Rating Inventory of Executive Function—Adult Version; ^11^ EHQ-21—Eating Habits Questionnaire-21; ^12^ EDI-3—Eating Disorder Inventory-3; ^13^ BSI—Brief Symptom Inventory; ^14^ DSQ-40—Defense Style Questionnaire-40, ^15^ F-DOS—Düsseldorf Orthorexie Scale; ^16^ TCI-56—Temperament and Character Inventory-56; ^17^ Ortho-11-Hu—Orthorexia Nervosa Questionnaire in Hungarian version (11 items); ^18^ AN—anorexia nervosa; ^19^ BN—bulimia nervosa, ^20^ ORTO-15—Orthorexia Nervosa Screening Tool; ^21^ MPS—Multidimensional Perfectionism Scale; ^22^ MBSRQ-AS—Multidimensional Body-Self Relations Questionnaire—Appearance Scale; ^23^ RSQ—Relationship Scales Questionnaire; ^24^ RSES—Rosenberg Self-Esteem Scale; ^25^ CFA—confirmatory factor analysis; ^26^ ORTO-11-ES—Orthorexia Nervosa Questionnaire (Spanish version, 11 items); ^27^ EDI-2—Eating Disorder Inventory-2; ^28^CFI—comparative fit index; ^29^TLI—Tucker–Lewis Index; ^30^ RMSEA—root mean square error of approximation; ^31^ TAS-20—Toronto Alexithymia Scale; ^32^ DERS-16—Difficulties in Emotion Regulation Scale-16; ^33^ SAAS—Social Appearance Anxiety Scale; ^34^ EAI—Exercise Addiction Inventory; ^35^ OSC—Obsessive–Compulsive Scale; ^36^ MOCI—Maudsley Obsessive–Compulsive Inventory; ^37^ BDI-II—Beck Depression Inventory-II; ^38^ BDDQ—Body Dysmorphic Disorder Questionnaire; ^39^ OCI-R—Obsessive–Compulsive Inventory—Revised; ^40^ BPPPS—Body Perception and Physical Performance Scale; ^41^ FFQ-6—Food Frequency Questionnaire-6; ^42^ EAT-40—Eating Attitudes Test-40.

### 3.4. Risk Factors in Adolescents and Young Adults

Additionally, it is important to pay attention to children and teenagers who suffer from orthorexia nervosa. Adolescence and early youth have a high demand for nutrients and microelements from food. It is important to ensure that these ingredients are supplied in the proper quantities in order to maintain optimal health on a somatic, cognitive, and emotional level. Adolescence is characterized by excessive criticism related to one’s own body and peer pressure regarding weight and body appearance, which can lead to the development of an obsession with healthy eating [52].

Some authors have noted a link between social media and the incidence of ON in adolescents, who are a more susceptible age group to manipulative influences in this regard. In one study of 680 young adults, Turner et al. noted that as many as 49% of the subjects suffered from ON (Table 3). The results may suggest that images of healthy meals posted on social media have more power to influence memory than verbal content, which may lead to an increased risk of orthorexia nervosa [6]. The occurrence of eating disorders in the adolescent population may be correlated with the development of orthorexia nervosa, as highlighted by other researchers [53]. Young men between the ages of 18 and 25 who are excessively focused on figure maintenance and restrictive dieting are a group at increased risk of developing orthorexia nervosa [54]. Interestingly, the correlation between the choice of nutrition-related majors, such as dietetics, and the propensity for orthorexia nervosa has also been recognized by researchers, who have shown an elevated tendency to develop the condition among students in these majors [7,8]. Also, the risk of orthorexia nervosa is higher in young adults who are engaged in healthcare professions as well as athletes and fitness enthusiasts [9,55]. It has also been linked to the co-occurrence of eating disorders and the fear of gaining too much weight, which is another risk factor [56]. Studies have suggested that excessive parental control can lead to anxiety that promotes the development of orthorexia nervosa, which was confirmed in 18% of patients studied [12]. It is also worth noting those who attend sports schools may be at higher risk of developing the disorder. Hyrnik and colleagues showed that orthorexia nervosa occurs with equal frequency in both children and adults, highlighting the importance of early diagnosis and intervention to prevent serious health consequences [57].

### 3.5. Potential Treatments

Although orthorexia is becoming increasingly recognized, there is still a lack of research on the effectiveness of various intervention methods and the long-term effects of actions taken in the treatment of this disorder. It is important to analyze how ON develops over time and what therapeutic strategies can be used to help in the rapid treatment of this disorder. Cognitive behavioral therapy (CBT), mindfulness-based techniques, and family therapy seem to be promising. Research on pharmacotherapy is also needed, especially in the case of comorbid anxiety or depressive disorders. The current lack of unified diagnostic and therapeutic guidelines limits the possibilities of effective intervention, which emphasizes the need for further research on the treatment of orthorexia.

## 4. Discussion

The aim of this study is to thoroughly investigate the factors leading to the development of orthorexia nervosa (OC) and to identify evidence supporting that OC belongs to the spectrum of eating disorders.

Orthorexia nervosa (OC) is characterized by an obsession with healthy eating. Nowadays, promoting healthy eating habits is usually recommended, but OC occurs when this behavior becomes too perfectionistic. There is an ongoing debate in the literature on two key clinical issues. The first one is whether OC (obsessive–compulsive symptoms) should be considered a behavioral phenomenon or lifestyle, or rather a mental disorder. Therefore, it is important to deepen the knowledge on how OC manifests itself in different populations and to develop therapeutic methods that will take into account both the specific symptoms of OC and interactions with other disorders [58].

### 4.1. Comorbidities

Many researchers point out that eating disorders such as anorexia nervosa (AN), bulimia nervosa (BN), and other eating disorders significantly increase the risk of developing orthorexia. In a study by Segura-Garcia et al., 32 patients with AN or BN were assessed using the ORTO-15 scale, the Yale–Brown–Cornell Eating Disorder Scale (YBC-EDS), and the Eating Attitude Test (EAT-26), both before and three years after treatment. The results showed a significant increase in ON symptoms after treatment in 58% of patients, suggesting that ON may develop in response to clinical improvement in eating disorders [14]. Additionally, studies suggest that the risk of ON may be particularly high in groups practicing yoga, where a greater tendency to orthorexic behavior is observed [11]. Furthermore, Kiss-Leizer et al. showed that factors characteristic of AN, BN, and OCD may be key parameters in the development of ON [13]. The comorbidity of ON with anorexia and obsessive–compulsive disorder (OCD) indicates the complexity of mechanisms that may affect the treatment of these patients. The overlap of these disorders requires tailored treatment strategies that not only address the specific symptoms of ON, but also take into account the interactions with symptoms of anorexia and OCD. Treatment of ON in people with AN and OCD should focus on developing more flexible eating habits and reducing anxiety related to deviations from “ideal” eating rules.

### 4.2. Body Image and Eating Habits

In addition to comorbidities, body image also plays a key role in the development of orthorexia, as it often forms the basis for the creation of unhealthy eating patterns. A study conducted at the University of New South Wales in Sydney revealed a significant association between body image and the occurrence of ON among Australian adults. In this study, the ORTO-15 tool for the diagnosis of ON was used, which indicated that 21% of participants showed features of orthorexia nervosa based on the adopted diagnostic threshold. Analysis of the results suggests that negative body image may promote the development of orthorexia nervosa, in which the obsession with healthy eating is used as a control strategy for body image [46]. Other researchers have observed similar correlations, indicating significant links between body image and the development of orthorexia nervosa (ON) [29,30,31]. Interestingly, both negative body image and excessive exercise may reinforce each other, leading to the exacerbation of orthorexia symptoms, especially in the context of ON. Excessive physical exercise often leads to obsessive focus on body image, which can deepen the problems associated with orthorexia nervosa (ON). Studies suggest that intensive exercise can reinforce unhealthy attitudes towards one’s body shape, which in turn contributes to the intensification of orthorexia nervosa symptoms [23,24,34,56].

In addition to the physical aspects of orthorexia, psychological factors also play a key role in the development of the disorder, deepening problematic habits. An interesting perspective is also presented a study on the relationship between orthorexia nervosa and psychopathological features. In the study, Noebel et al. examined the occurrence of orthorexia nervosa in 405 people suffering from problems with cognitive functions using the Orthorexia Nervosa Inventory (ONI) and the Behavioral Assessment of Executive Functions for Adults (BRIEF-A). The results indicated links between the level of orthorexia nervosa and deficits in executive functions, especially in the area of behavioral regulation, such as emotion control, inhibition, and self-control [22]. Similar results were obtained by other researchers focusing on psychopathology in the sense of ON tendencies. After conducting research involving students at the University of Palermo, they found that people diagnosed with ON had greater psychopathological symptoms than other groups [24]. An interesting reference is also a study conducted on 3235 students with personality disorders. The results obtained suggest that ON correlates with psychopathological features in young adults [27]. Other researchers have noticed similar links [17,20]. Other studies focus on examining the personality profile of people with a tendency to orthorexia nervosa. After the participants completed an online survey, it was concluded that people with a tendency to avoid risk and threats are at greater risk of ON [20].

When analyzing publications on orthorexia nervosa, it is important to pay attention to eating habits, which are a key element in research on this disorder. The literature increasingly emphasizes the link between specific eating habits and the occurrence of orthorexia nervosa. In the study conducted by Anna Dittfeld and co-authors, the relationship between vegetarianism and orthorexia nervosa was analyzed. The study included a sample of 2611 participants, including 1346 vegetarians and 1265 non-vegetarians. The study used a questionnaire that included, among others, the BOT. The results suggest that vegetarians may be more susceptible to the development of orthorexia nervosa, which indicates clear differences in the frequency of this disorder depending on the type of diet [36]. Similar correlations were also noted in the study by Kalik et al. [48]. The participants of another study were students at the University of Pavia. The analysis involved 671 students (including dietetics, economics, and humanities). Although no significant differences in the risk of ON were found between students of different fields of study, the diet used was confirmed as the main risk factor for orthorexia nervosa [37]. ON is increasingly diagnosed in young adults, which is why other researchers also pay attention to the study of the relationship between eating style and ON. In the population of students at an Italian university, the type of diet used turned out to be a predictor of ON [47]. Voglino et al. examined customers of organic shops (OSC). The ORTO-15 test and the Eating Habits Questionnaire (EHQ) were used as questionnaires to assess sociodemographic characteristics and current eating habits. Interestingly, the results of the study showed that customers of organic shops were more likely to obtain positive results in the ORTO-15 test compared to people from the non-OSC group. The prevalence of ON symptoms among OSC was 69.4% using 40 cut-off points and 23.1% using 35 cut-off points in ORTO-15 [38]. Interestingly, “eating with awareness” within mindful eating (ME) has a negative association with ON, suggesting that people who are more aware of their eating habits may be less prone to orthorexia nervosa [49]. Many researchers are attempting to standardize the results of studies to better understand how different BMI levels affect the risk of developing orthorexia nervosa [14]. One large study conducted on a group of 864 people showed that people with a higher BMI are more likely to develop orthorexia nervosa [14]. This is confirmed by the results obtained by Bundros et al., where overweight people achieved higher scores in the Bratman Test (BOT) [32]. In the literature, there are studies on the correlation of high BMI and the occurrence of ON [12]. However, other studies indicate the opposite trend, with an association between low BMI and ON [24,33]. In the study by Karniej et al. conducted in Poland and Spain, unique predictors of psychological orthorexia nervosa (ON) were identified among homosexual men. Interestingly, low body mass index (BMI) has been shown to be a significant predictor [34]. Analysis of the studies indicates that both high and low body mass index (BMI) may affect the risk of developing orthorexia nervosa (ON), but the results are ambiguous and varied, which emphasizes the need for further research to fully understand these relationships. The discrepancies that occur may be due to methodological differences, such as different definitions and diagnostic criteria for ON used in different studies. Cultural differences are also an important factor, influencing the perception of health, nutrition, and BMI in individual populations. These differences shape specific attitudes towards health, which may vary significantly across cultures, which influences the way BMI is perceived. These attitudes may be reinforced by social media, which promote global patterns of beauty and healthy lifestyles, influencing eating habits.

Additionally, differences in the demographics of the study groups, such as age, gender, socioeconomic status, or geographic location, may shape the varied research results. It is also worth noting that the lack of standardization of diagnostic tools used to diagnose ON may overestimate the scale of the problem. This is evident in a study on a group of students from Sydney. When reviewing the literature on orthorexia nervosa (ONER), it can be seen that many studies focus on the role of gender as a significant factor influencing orthodox tendencies. These publications often indicate differences in the prevalence of ONER between women and men, with results suggesting that women may be more vulnerable to developing ONER than men. In the Dell’Osso study, 2130 participants were examined who were students at one of the universities in Italy. The results indicate that ONER, characterized by a pathological approach to eating related to concerns about health and food, is more common among women [47].

### 4.3. The Influence of Social Media

In today’s digital world, eating habits are largely shaped by social media, which promotes specific diets and lifestyles that can further amplify orthorexia symptoms. These reports are now prompting researchers to conduct a detailed analysis of its prevalence in younger age groups, including children and young adults. Among adolescents, it is crucial to understand how different factors may influence the development of this disorder. In one study, Turner et al. drew attention to the influence of social media on the incidence of orthorexia nervosa, finding that intensive use of platforms such as Instagram was associated with a higher risk of its occurrence. The results suggest that users who actively follow accounts related to healthy eating may be more prone to obsession with healthy eating. In this study, the incidence of this disorder was 49% [59]. In young adults, a correlation between the occurrence of eating disorders and ON can also be observed [55]. The White Mika study involved 103 male university students who completed an online survey assessing the symptoms of orthorexia nervosa. After analyzing the obtained results, the authors noted associations between ON and eating disorders occurring with exercise addiction [53]. An interesting approach is to consider ON in the context of parental control. In a study of 403 Italian youth athletes (231 boys and 172 girls) aged 14–18 years, it was shown that parental psychological control has a negative impact on children’s emotional states, which is associated with a higher incidence of ON [56]. In the preliminary study, 116 student athletes and 99 non-athletes from the North East England were conducted to assess the incidence of ON in this age group. The ORTO-15 test was used for the assessment. The results showed a high incidence of ON symptoms, reaching 76% among all participants [12]. In turn, a study of 1899 patients attending secondary schools revealed that the main factors associated with orthorexia nervosa in young people are overweight, intense sports activity, involvement in extracurricular activities, smoking, working parents, and high family income. These factors influence the development of orthorexia nervosa in this age group, shaping their approach to healthy eating and diet-related behaviors. The mean score in the ORTO-15 test was 39.2 ± 3.6 points, without taking into account gender differences [57]. Studies on orthorexia nervosa (ON) in the younger population were conducted among medical students at the University of Inu. Using the ORTO-15 test, it was found that 76.2% of participants showed orthorexia nervosa tendencies [7]. Similar associations between ON and studying a medical profession have been observed in other studies [8,54]. Studies on orthorexia nervosa in the younger population reveal a number of significant associations with various risk factors, which emphasizes the complexity and multifaceted nature of this disorder. Contemporary studies on ON increasingly focus on the influence of social media on its occurrence. Social media, such as Instagram and Facebook, are seen as tools that can shape attitudes and behaviors related to nutrition not only in adolescents but also in adults. Mass media often promote unrealistic body patterns, which can lead to comparisons and dissatisfaction with one’s own appearance. Additionally, easy access to the so-called “experts” and dietary challenges online may promote unhealthy eating practices that may worsen ON symptoms.

The results of a study conducted in Germany indicate a link between following accounts related to health and physical fitness and orthorexia nervosa tendencies. This study was conducted on a group of adult women and men aged 18–30 [60]. Other authors have also noted that the use of social media, especially accounts related to healthy eating on Instagram, is associated with a high incidence of orthorexia nervosa symptoms, which is particularly visible in young people. One study examined 653 teenagers and showed that young people with strong symptoms of social media addiction suffer from a higher risk of developing eating disorders (OR = 2.04, 95% CI 1.34–3.12) [61].

The influence of social media on the development of orthorexia may be particularly pronounced across cultural boundaries, where social norms and expectations regarding the body and health may differ significantly.

### 4.4. Cultural Differences and Profession

Interestingly, the results of studies conducted in countries with large cultural differences, such as Russia and Spain, show significant differences in the way different social groups react to the influence of social media. A study conducted in Russia shows that Instagram users who post orthorexia nervosa-related content have different experiences. Positive content can sometimes support a healthy relationship with food, while negative content can increase obsessions with healthy eating. In Spain, on the other hand, intensive use of various social media platforms was associated with a higher risk of eating disorders [61]. Similar results were obtained by Gramaglia et al., in which

Perfectionism, which may be reinforced by idealized body images presented in social media, is also a significant risk factor for orthorexia nervosa [19,42]. Studies conducted among medical and nursing candidates reveal that people in these professions, where taking care of health and body appearance plays a key role, are more likely to develop orthorexia nervosa. High levels of social media addiction and orthorexic tendencies are particularly evident in this group, suggesting that pressure related to professional image and perfectionism may reinforce orthorexia nervosa tendencies [62]. Other studies also increasingly indicate the occurrence of orthorexia nervosa (ON) among medical professionals, especially in professions related to nutrition. Studies suggest that people studying or working in fields related to dietetics, such as dietitians, nurses, and medical students, may be at greater risk of developing ON. Interestingly, studies on orthorexia nervosa (ON) have been conducted in different parts of the world, such as Australia, New Zealand, and the United States, indicating that the problem of ON among medical professions is of a global nature. The fact that studies from different environments lead to similar conclusions suggests that there is a universal correlation between ON and the abovementioned factors, regardless of the region in which the studies are conducted [59,63].

It is worth noting that the studies discussed provide a lot of valuable information that can be helpful in diagnosing ON. However, their limitations should be noted. Many of the studies cited are based on diagnostic tools that are not standardized, which may affect the discrepancies in the obtained results. In addition, most studies were conducted on specific populations, which limits the possibility of generalizing the results to wider groups. Factors such as cultural, demographic, or social differences may have a significant impact on the results, which requires further research on more diverse groups. However, the strengths of the existing studies are their interdisciplinary nature, combining various aspects of mental and physical health, and showing the complexity of orthorexia. To fully understand the mechanisms associated with ON, further, more diverse studies that will take into account these limitations and use more solid research methods are necessary.

### 4.5. Limitations

The analyzed publications point to several important limitations. These include the lack of standardized diagnostic tools for assessing orthorexia (ON) and inconsistency in defining the disorder. The use of a diagnostic tool such as the ORTO-15 can produce hypocritical test results. The reason for this is the varying number of cut-off points used by researchers—some studies use a threshold of 35 points, others 40 or 45 [9].

In the absence of standardization, a diagnostic tool such as ORTO-15 can lead to an incorrect diagnosis. For example, an overestimation of the scores obtained may result in the unnecessary implementation of dietary or psychological interventions for patients. Conversely, under-scoring and overlooking cases of ON may involve overlooking a health problem, which can also have serious consequences. Therefore, it is important to introduce and validate standardized diagnostic tools to minimize the risk of misdiagnosis.

Another major limitation is understanding whether ON represents an independent clinical disorder, whether it belongs to the eating disorder spectrum, or whether it is a form of behavioral addiction or a variant of obsessive–compulsive disorder. The lack of consensus on the definition of ON hinders further research and clinical practice.

It is also worth noting the need for longitudinal studies of ON that could assess how the disorder co-occurs with other problems, such as anorexia or bulimia, as well as what role sociocultural factors, such as the promotion of healthy lifestyles in the media, play. Studies of this kind could determine whether ON is distinct or an outgrowth of other disorders, as well as the long-term effects of the disorder.

Understanding and effectively treating orthorexia requires interdisciplinary collaboration between mental health professionals and nutritionists

## 5. Conclusions

The obtained research results indicate significant associations between orthorexia (ON) and eating disorders (ED). The highest risk of ON is observed in individuals focused on substantial weight loss, displaying perfectionism, dissatisfaction with body image, excessive exercise, and difficulties in interpersonal relationships. The analysis also revealed significant correlations between ON, body mass index (BMI), and gender; however, no significant relationship was found between ON and obsessive–compulsive disorder (OCD).

Based on available studies, orthorexia can be considered a disorder that falls within the spectrum of eating disorders, though it should not be classified as a classic subtype of anorexia or a pure form of OCD. Therefore, we suggest that ON be treated as a distinct disorder within the “Eating Disorders” category in classifications such as the DSM or ICD. Such classification would allow for the precise differentiation of ON from anorexia and OCD and facilitate the development of more tailored diagnostic and therapeutic methods. Including ON in psychiatric classifications such as the DSM or ICD would also aid in accurate diagnosis and effective therapeutic interventions.

The literature review suggests that a promising direction for future research is to investigate the correlation between orthorexia and excessive engagement with social media, addiction to it, and the health status of students in health-related fields. The high risk of ON in these groups has already been noted in the literature [6,8,50,51,62,64,65,66,67,68,69]. Future research should also focus on assessing the effectiveness of various therapeutic strategies for treating ON. Additionally, raising awareness and educational efforts regarding orthorexia, both among the general public and healthcare professionals, are crucial in minimizing the long-term effects of this disorder.

## Figures and Tables

**Table 1:** Linking ON to nutrition-related abnormalities.

No.	Researcher	Purpose of the Survey	Research Methods	Study Group		Frequency	Results and Comments
				Number of Participants	Group Characteristics		
1	[13]	How common ON ^1^ is and its interrelation with ED ^2^ and OCD ^3^	ORTO-15 ^4^, EAT-26 ^5^, MOCI ^6^, individual questionnaire	864	599 women aged 17 to 23 and 265 men aged 15 to 21	27%, a reading of 35 was regarded to be the cut-off point	ED ^2^ increases risk of ON ^1^; high BMI contributes to ON ^1^. The greatest danger was noted for those aged 13–16 and the smallest for those between 16–19
2	[15]	Frequency of appearance ON ^1^ levels in women diagnosed with AN ^7^ and BN ^8^	ORTO-15 ^4^, YBC-EDS ^9^, Eat-26 ^5^	32	Females with a diagnosis of anorexia (AN ^7^) or bulimia (BN ^8^).	High prevalence of ON ^1^ among female patients with AN ^7^ and BN ^8^. The treatment was positive in the ORTO-15 ^4^ test, which proved to be higher (28%) and (58%) than the control group, in which it was merely 6%.	AN ^7^ and BN ^8^ with a tendency towards ON ^1^
3	[16]	Occurrence of similarities and dissimilarities between ON ^1^ and ED ^2^ in patients with AN ^7^ and BN ^8^	ORTO-15 ^4^, EAT-26 ^5^, MDBSRQ ^10^	577	Female ED ^2^ patients	Prevalence of ON ^1^ is notably high in women with AN ^7^ and BN ^8^. High performances on the ORTO-15 ^4^ (28%) and EAT-26 ^5^ (14%) were observed in athletes.	AN ^7^ and BN ^8^ increase the likelihood of ON ^1^
4	[17]	Comparison of ON ^1^ and AN ^7^ between women with AN ^7^ and patients without diagnosed diseases	ORTO-15 ^4^	136	AN ^7^ patients from Italy and Poland	High prevalence of ON ^1^ among women with AN ^7^. In the Italian control group studied, 54% of participants scored 40 or more on the ORTO-15 ^4^ test. 46% of this group may have orthorexic behavior	AN ^7^ predisposed to ON ^1^. Also, significant differences were detected between the Italian and Polish samples in the frequency of orthorexia nervosa and ORTO-15 ^4^ scores, suggesting cultural differences requiring further investigation
5	[18]	Prevalence of ON ^1^ in students; a study of the relationship between ON ^1^ and psychological attitudes	ORTO-11-ES ^11^ Eating Disorder Inventory (EDI-2) ^12^	492	The research group consisted of students from the University of Castilla-La Mancha in Spain (280 young women and 212 men)	The confirmatory factor analysis (CFA) identified 11 items and 3 attributes as the superior matching model; CFI ^13^ = 0.94, TLI ^14^ = 0.91, RMSEA ^15^ = 0.058	Many behaviors resulting from ED ^2^ correlate with ON ^1^. ON ^1^ is more common in the female gender.
6	[19]	Observe the relationships between ON ^1^ and potential factors contributing to ON ^1^ in an international group of yoga practitioners	Teruel’s orthorexia nervosa scale, yoga immersion scale, passion scale, Frost’s perfectionism scale, NEO-PI-R self-discipline scale, EDI leanness scale, appearance beliefs scale	469	Yoga practitioners	Not determined	The pursuit of thinness was seen as a major risk factor for ON ^1^
7	[20]	Examined whether perfectionism, figure disorders, relationships with the environment, and self-esteem bias contribute to the prognosis of ON ^1^	OR ORTO-15 ^4^, MPS, MBSRQ-AS, RSQ ^16^, RSES ^17^	220	The survey group was 46 men and 174 women; 180 of those were first- and second-year psychology students at James Cook University	The mean score for ORTO-15 (based on nine items) was 22.71 (SD = 4.55). There was no significant difference in ORTO-15 ^4^ scores between men and women	ON ^1^ correlates strongly with perfectionism, feelings of body satisfaction, and attachment style, especially in those with ED ^2^ present
8	[21]	The aim was to investigate the personality profile of people with a high orthorexic tendency	Temperament Character Inventory-56 (TCI-56), Ortho-11-Hu ^18^.	739	Participants’ ages ranged from 18 to 72 years. The research group consisted of doctors, athletes, nutritionists, performance artists, and Ashtanga yoga practitioners	The mean ORTO-11-Hu ^18^ score was 22.71 (SD = 4.55). The high ON ^1^ tendency group (score 14–29) comprised 234 subjects and the low ON ^1^ tendency group (score 35–43) comprised 245 subjects. Women were more likely than men to show high ON ^1^ tendencies	The psychological factors describe as one of the key symptoms of orthorexia nervosa may be its association with risk factors for AN ^7^ and BN ^8^
9	[22]	To explore whether perfectionism and self-perception contribute to ON ^1^ symptoms in people with misconceptions about healthy eating	12-item Clinical Perfectionism Questionnaire, 20-item Beliefs About Appearance Scale (BAAS), Orthorexia Nervosa Inventory (ONI)	456	The group consisted of 165 males and 287 females (4 reported gender as non-binary) ranging in age from 19 to 77 years old	In the table showing descriptive statistics and correlations, the frequency of orthorexia nervosa (ON ^1^) in the sample is 4.8% (*n* = 22)	Perfectionism is indirectly associated with ON ^1^ symptoms through health-focused self-awareness
10	[23]	The analyses examined whether the prevalence of orthorexia nervosa is associated with executive functioning disorders	Orthorexia Nervosa Inventory (ONI), BRIEF-A ^19^	405	Among participants, 80% were female. Half Caucasian. Average age = 24 years; average BM ^20^ I = 25	The prevalence of orthorexia nervosa (ON ^1^) symptoms was weakly to moderately correlated with all BRIEF-A ^19^ scales	In addition to their unique symptoms, orthorexia nervosa and anorexia may share similar neuropsychological characteristics
11	[24]	This study examined how scores on the SPAS ^21^ and the SATAQ ^22^ questionnaire relate to BOT ^23^ scores in relation to age, gender, and exercise durations	Sociocultural Attitudes Towards Appearance Questionnaire (SATAQ) ^22^ relate to Bratman’s orthorexia nervosa test (BOT) ^23^	251	The group was made up of 85 men and 166 women. The age range was 17–62 years for men and 34 years for women. Most of the men (66%) and half of the women (50%) practiced between three to four times a week	Frequency of orthorexia nervosa (ON ^1^) was significantly correlated with internalization of the SATAQ subdomain in men (β = 0.14, *p* = 0.019). In women, the strongest factors were SPAS scores (β = 0.28, *p* < 0.0001) and frequency of exercise (β = 0.88, *p* < 0.0001)	Fitness center participants who exercised had high BOT ^23^ scores. Excessive exercise with pathological weight control woods the occurrence of pathological behavior and ED ^2^
12	[25]	Investigate the associations between ON ^1^ and the core features of eating disorders (EDs), psychopathological symptoms, and defense mechanisms	ON ^1^ (EHQ-21) ^24^, eating psychopathology (EDI-3) ^25^, psychopathological symptoms (BSI) ^26^, and defense mechanisms (DSQ-40) ^27^	270	Students from the University of Palermo in southern Italy and were assigned to three groups: 52 people with ON ^1^ symptoms, 157 people constituted a healthy eating control group and 61 people following a normal diet	Not specified	Group with ON ^1^ symptoms reported more ED ^2^ features, more psychopathological symptoms

^1^ ON—orthorexia nervosa; ^2^ ED—eating disorder; ^3^ OCD—obsessive–compulsive disorder; ^4^ ORTO-15—the Bratman Orthorexia Test; ^5^ EAT-26—Eating Attitudes Test; ^6^ MOCI—Maudsley Obsessive–Compulsive Inventory; ^7^ AN—anorexia nervosa; ^8^ BN—bulimia nervosa; ^9^ YBC-EDS—Yale–Brown Obsessive–Compulsive Scale for Eating Disorders; ^10^ MDBSRQ—Multidimensional Body–Self-Relations Questionnaire; ^11^ ORTO-11-ES—Spanish version of the 11-item orthorexia scale; ^12^ EDI-2—Eating Disorder Inventory; ^13^ CFI—Comparative Fit Index; ^14^ TLI—Tucker–Lewis Index, a measure of model fit; ^15^ RMSEA—root mean square error of approximation; ^16^ RSQ—Relationship Scales Questionnaire; ^17^ RSES—Rosenberg Self-Esteem Scale; ^18^ Ortho-11-Hu—11-item Orthorexia Questionnaire (Hungarian version); ^19^ BRIEF-A—Behavior Rating Inventory of Executive Function—Adult Version; ^20^ BMI—body mass index; ^21^ SPAS—Social Physique Anxiety Scale; ^22^ SATAQ—Sociocultural Attitudes Towards Appearance Questionnaire; ^23^ BOT—Bratman Orthorexia Test; ^24^ EHQ-21—21-item Eating Habits Questionnaire; ^25^ EDI-3—Eating Disorder Inventory; ^26^ BSI—Brief Symptom Inventory; ^27^ DSQ-40—Defense Style Questionnaire.

**Table 3:** Orthorexia nervosa in children and young adults.

No.	Researcher	Purpose of the Survey	Research Methods	Study Group		Frequency	Results and Comments
				Number of Participants	Group Characteristics		
1	[6]	Impact of social media on ON ^1^ incidence in young adults	ORTO-15 ^2^, web poll	680	The study group consisted of 23 men, 686 women, and 4 people identifying themselves as other	In the study group, mental orthorexia nervosa was present in 49% of the participants, a significantly higher rate compared to the general population, where it is less than 1%.	Risk of ON ^1^ increases with frequent use of Instagram
2	[53]	Correlation of orthorexia using SuperMimic software for healthy lifestyle and physical activity addiction	Internet survey Qualtrics ^3^, ORTO-7 ^4^	113	Undergraduate students aged 18–25 attending a large, private university in the northeastern United States	Relationship between ON ^1^ and ED ^5^ symptoms in most subjects	ON with high right-sidedness may occur in young men with ED
3	[55]	Determining the frequency of orthorexia nervosa in nursing students and the correlation between ED ^5^ and ON ^1^	ORTO-11 ^6^, EAT-40 ^7^,	181	Nursing students	45.3%	Nearly half of nursing students in residency suffering from ED ^5^ are at risk of having ON ^1^
4	[56]	Impact of excessive parental control on the incidence of ON ^1^ in children during adolescence	ORTO-15 ^2^ DAPCS ^8^	403	The participants were Italian teenage athletes (231 boys and 172 girls) aged between 14 and 18	Addiction-oriented control was linked to depression (b = 0.26; *p* < 0.001) and anxiety (b = 0.40; *p* < 0.001), while achievement-oriented control predicted depression (b = 0.28; *p* < 0.001) and anxiety (b = 0.32; *p* < 0.001)	Too much parental control can cause anxiety, leading to ON ^1^ incidences
5	[12]	Investigating the frequency of orthorexia nervosa in young athletes	ORTO-15 ^2^	116	The study group consisted of students with an average age of 21 ± 1 years and 99 non-athletes (21 ± 2) from universities in North East England who completed the ORTO-15 ^2^ test, with a cut-off value of ≤40	Symptoms of orthorexia nervosa were present in 76% of students. There was no significant difference in ORTO-15 ^2^ scores between athletes (36.6 ± 3.9) and non-athletic students (37.2 ± 3.8; *p* = 0.279)	ON may occur in young students
6	[57]	Assessment of the prevalence of ON ^1^ in adolescents living in Poland	ORTO-15 ^2^	1899	The subjects were 992 girls and 907 boys aged 15 to 21 years old	The ORTO-15 ^2^ value averaged 39.2 ± 3.6 points, with no gender difference	The incidence of ON ^1^ is similar to that estimated in adults
7	[7]	Analysis of the prevalence of orthorexia nervosa in medical students	ORTO-15 ^2^, EAT-40 ^7^	298	Students of the Faculty of Medicine of the University of Inonu in 2017	The prevalence of orthorexia nervosa among students was 76.2%, while 11.1% of students struggled with an eating disorder	The study postulates that health workers are more prone to orthorexia nervosa than other groups
8	[54]	The aim of this study is to investigate the correlation between ON and ED, body composition	EAT-40 ^7^ ORTO-11 ^6^ anthropometric measurements	136	Participants were female students who had scores on the Orthorexia nervosa Psychological Questionnaire (ORTO-11) ^6^ and the Eating Attitudes Test (EAT-40) ^7^ indicating the presence of orthorexia nervosa	The overwhelming proportion of participants (70.6%) obtained high scores on the ORTO-11 ^6^ test	Female nutrition and dietetics students showed orthorexia nervosa tendencies
9	[8]	Investigating the association of ON ^1^ prevalence in dietetics students	ORTO-15 ^2^	440	The study group consisted of students from three different schools: 53 from the dietetic school, 200 from the exercise and sports science school, and 187 from the biology school	All schools showed a high prevalence of orthorexia nervosa traits: 35.9% in the dietetic school, 26.5% in the biology school, and 22.5% in the exercise and sports science school	There is a clear prevalence of orthorexia nervosa among dietetics students

^1^ ON—orthorexia nervosa; ^2^ ORTO-15—Orthorexia Nervosa Test (15-item version); ^3^ Qualtrics—An online survey software platform; ^4^ ORTO-7—Orthorexia Nervosa Test (7-item version); ^5^ ED—eating disorder; ^6^ ORTO-11—Orthorexia Nervosa Test (11-item version); ^7^ EAT-40—Eating Attitudes Test (40-item version); ^8^ DAPCS—Dysfunctional Attitudes about Parenting and Child Socialization.
